# Nomogram for predicting overall survival in stage II‐III colorectal cancer

**DOI:** 10.1002/cam4.2896

**Published:** 2020-02-06

**Authors:** Jungang Liu, Xiaoliang Huang, Wenkang Yang, Chan Li, Zhengtian Li, Chuqiao Zhang, Shaomei Chen, Guo Wu, Weishun Xie, Chunyin Wei, Chao Tian, Lingxu Huang, Franco Jeen, Xianwei Mo, Weizhong Tang

**Affiliations:** ^1^ Department of Gastrointestinal Surgery Division of Colorectal & Anal Surgery Guangxi Medical University Cancer Hospital Nanning Guangxi Zhuang Autonomous Region P.R. China; ^2^ Guangxi Clinical Research Center for Colorectal Cancer Nanning Guangxi Zhuang Autonomous Region P.R. China; ^3^ Collaborative Innovation Center for Targeting Tumor Diagnosis and Therapy Guangxi Medical University Nanning Guangxi Zhuang Autonomous Region P.R. China

**Keywords:** colorectal cancer, nomogram, prognosis, survival

## Abstract

**Purpose:**

The overall survival (OS) of patients diagnosed with stage II‐III colorectal cancer (CRC) can vary greatly, even between patients with the same tumor stage. We aimed to design a nomogram to predict OS in resected, stage II‐III CRC and stratify patients with CRC into different risk groups.

**Patients and Methods:**

Based on data from 873 patients with CRC, we used univariate Cox regression analysis to select the significant prognostic features, which were subjected to the least absolute shrinkage and selection operator (LASSO) regression algorithm for feature selection. Cross‐validation was used to confirm suitable tuning parameters (λ) for LASSO logistic regression. Then, the nomogram was used to estimate 3‐ and 5‐year OS based on the multivariable Cox regression model. The survival curves of the two groups were produced using the Kaplan‐Meier method. Risk group stratification was performed to assess the predictive capacity of the nomogram.

**Results:**

Preoperative mean platelet volume, preoperative platelet distribution width, monocytes, and postoperative adjuvant chemotherapy were identified as independent prognostic factors by LASSO regression and integrated for the construction of the nomogram. The nomogram provided good discrimination, with C‐indices of 0.67 and 0.69 for the training and validation sets, respectively. Calibration plots illustrated excellent agreement between the nomogram predictions and actual observations for 3‐ and 5‐year OS. Moreover, a significant difference in OS was shown between patients stratified into different risk groups (*P* < .001).

**Conclusion:**

We constructed and validated an original predictive nomogram for OS in patients with CRC after surgery, facilitating physicians to appraise the individual survival of postoperative patients accurately and identify high‐risk patients who need more aggressive treatment and follow‐up strategies.

## Graphical abstract

Four points are allocated for preoperative mean platelet volume, preoperative platelet distribution width, monocytes, and postoperative adjuvant chemotherapy. Nomogram for predicting 3‐ and 5‐year probabilities of colorectal cancer patients was established. Draw a vertical straight line from the variable value to the axis labeled “Points”. Then calculate all variables’ points. The total points on the bottom scales that correspond to the 3‐ and 5‐year survival were showed apparently.

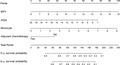

## 1 INTRODUCTION

Colorectal cancer (CRC) is one of the most common malignancies, with high incidence and mortality rates, and ranks as the third most frequent cancer and the second leading cause of cancer‐related deaths worldwide.^1^ In China, CRC is the fifth most common cause of cancer‐related deaths (8.6%).^2^ For patients with stage II‐III CRC, the standard, potentially curative treatment is radical resection and neoadjuvant chemoradiotherapy.^3^, ^4^ Despite recent advances in chemotherapy, its clinical effect is not ideal, as indicated by local recurrence and distant metastasis rates.^5^ Even with radical resection and neoadjuvant chemoradiotherapy, the 5‐year overall survival (OS) rates for stage II‐III CRC remain unsatisfactory, with 5‐year survival rates of approximately 70% for stage II and 60% for stage III.^6^

The TNM staging system of the seventh edition of the American Joint Committee on Cancer is widely used in prognosis prediction for patients with CRC.^7^ However, there are still some limitations in postoperative OS prediction. The prognosis of patients with CRC can vary greatly, even within the same TNM stage. The OS of patients with stage II‐III CRC is affected by many factors besides tumor stage, such as tumor location, histological type, age, sex, microsatellite status, and RAS/RAF mutation, and no single factor can accurately predict survival in CRC.^8^, ^9^ In addition, sophisticated and expensive laboratory techniques have limits to their application. Nomograms have been widely used in clinical oncology as reliable tools for estimating numerical probabilities for individual patients by incorporating and illustrating important prognostic factors.^10^, ^11^, ^12^, ^13^ This has been demonstrated in several types of cancers, including breast,^14^ lung,^15^ and gastric cancers,^12^ and the predictions of these nomograms may be more accurate than those based on the traditional TNM staging systems for various tumor types.^16^, ^17^ Furthermore, we have thus far found relatively few nomograms for predicting survival in patients with stage II‐III CRC.

In this study, we aimed to develop a prediction model for the OS of resected stage II‐III CRC that integrates all the identified significant prognostic factors and, further, to stratify patients into different risk groups. This nomogram will provide more individualized prognoses, which can help clinicians and patients make informed treatment and management decisions.

## 2 PATIENTS AND METHODS

### 2.1 Patients’ selection criteria

Ethical approval for this retrospective analysis was obtained from our institutional review board. On the basis of the appointed inclusion and exclusion criteria, 873 patients with stage II‐III CRC who underwent surgical treatment at Guangxi Medical University Cancer Hospital between June 2004 and October 2018 were included.

The inclusion criteria were as follows: (a) patients with CRC; (b) patients who underwent surgical resection of the primary tumor; (c) patients with II‐III pathological staging for CRC; The exclusion criteria were as follows: (a) patients who received antitumor therapy before surgery (including radiotherapy, chemotherapy, or chemoradiotherapy); (b) patients who had other malignancies in the same period; (c) patients with severe liver disease and/or acute infection; (d) patients with incomplete follow‐up data whose prognosis was unknown.

The medical records were obtained based on relevant demographic and clinical features. These features were divided into several categories, including general information, diagnosis, signs and symptoms, prior history, personal history, family history, auxiliary examination, imaging and endoscopy examination, pathological examination, and postoperative situation. General information, which was based on the patients’ identity cards, height, and weight were extracted from the hospital database. Diagnosis was based on the patients’ discharge diagnoses. Previous, personal, and family history were extracted from the hospital admission records, which were verified by a senior physician. Auxiliary examination was collected from tests performed on the first admission. Imaging and endoscopy examination were based on patients’ preoperative CT, MRI, and colonoscopy. Pathological examination included patients’ preoperative endoscopic biopsies and postoperative pathological reports. The names of 242 features in the study are listed in Table S1. Patients were followed‐up regularly according to the National Comprehensive Cancer Network guidelines. The primary endpoint was OS, which was defined as the time from surgery to death, regardless of the cause.

### 2.2 Feature selection

Features included in the nomogram were selected in two steps. First, univariate Cox regression analyses were conducted to screen for features significantly related to OS. Clinical features with a *P*‐value < .05 in the univariate analysis were selected. The selected features were then used in the least absolute shrinkage and selection operator (LASSO) regression algorithm. Dummy variables were created for categorical variables. Cross‐validation was used to confirm suitable tuning parameters (*λ*) for LASSO logistic regression. Finally, the most significant features were selected by LASSO.

Construction of the Nomogram.

The most significant features selected by LASSO from the training dataset were used for multivariate Cox proportional hazards analyses; variables with *P*‐values < .05 by multivariate analysis were incorporated into nomograms that were constructed to predict the 3‐ and 5‐year OS rates.

### 2.3 Validation of the nomogram

The concordance index (C‐index) was calculated with the Cox regression model method. The value of the C‐index was between 0.5 and 1.0, which indicates the decent discriminatory capacity of the nomogram, whereas 0.5 indicates a random chance. The C‐indices of two models (training set and validation set) were contrasted to assess the discrimination of the nomogram. The observed 3‐ and 5‐year OS were compared to the predicted 3‐ and 5‐year OS to further verify the predictive performance of the nomogram. We assessed the goodness‐of‐fit of the nomogram using calibration plots and a modified Nam‐D'Agostino test. The modified Nam‐D'Agostino test is an extension of the Hosmer‐Lemeshow goodness‐of‐fit test in survival data.^18^

### 2.4 Risk group stratification based on the nomogram

The risk scores of patients were calculated using the nomogram. To determine the optimal segmentation threshold for patient stratification, we repeatedly split the patients in the whole database into low‐risk and high‐risk groups based on each risk score. For each division, hypothesis testing was performed, and the hazard ratios (HR) and *P*‐values of the log‐rank test were recorded. The optimal segmentation threshold was determined when the minimum *P*‐value of the log‐rank test was obtained. Patients whose risk scores were higher than the optimal segmentation threshold were assigned to the high‐risk group, and the rest were assigned to the low‐risk group. The survival curves of the two groups were produced with the Kaplan‐Meier method.

### 2.5 Statistical analysis

R statistical software (version 3.4.0.) was used to perform the statistical analyses. LASSO regression analysis was operated with the “glmnet” package. *P*‐values < .05 indicated statistical significance.

## 3 RESULTS

### 3.1 Clinical characteristics

In total, 873 eligible patients with integrated information were incorporated in the dataset and randomly divided into two independent cohorts at a ratio of 6.5:3.5 (training cohort, n = 569; validation cohort, n = 304). There were 525 male patients and 348 female patients included in this study. The average age of these patients was 59 years. CT was performed an average of 6.2 days (range, 3‐10) before surgery. Most patients had T3‐4 stages (841 cases, 96.33%) and N0 stages (468 cases, 53.6%) based on preoperative CT. The demographic and clinical features of the patients in the training and validation sets are listed in Table 1.

**Table 1:** Patient background characteristics

Characteristics	Case n	Training set n (%)	Validation set n (%)	*X^2^*	*P*
Age					.083^a^
Median (IQR)(years)	873	60 (49,68)	61 (51,69)		
Sex				0.29	.86
Male	525	341 (59.9)	184 (60.5)		
Female	348	228 (40.1)	120 (39.5)		
pT Classification				2.45	.12
T1‐T2	32	25 (4.4)	7 (2.3)		
T3‐T4	841	544 (95.6)	297 (97.7)		
pN Classification				0.79	.78
N0	468	307 (54)	161 (53)		
N1‐N2	405	262 (46)	143 (47)		
Adjuvant Chemotherapy				0.12	.73
Yes	533	345 (60.6)	188 (188)		
No	340	224 (39.4)	116 (116)		
MPV					.57^a^
Median (IQR) (fL)		9.4 (8.6,10.2)	9.3 (8.5,10.1)		
PDW					.63^a^
Median (IQR) (fL)		15.4 (12.3,15.8)	15.4 (12.4,15.9)		
Monocytes					.54^a^
Median (IQR) (10^9^/L)		0.46 (0.35,0.57)	0.43 (0.34,0.56)		

Abbreviations: MPV, mean platelet volume; PDW, preoperative platelet distribution width.

a *t* test

### 3.2 Feature selection

**Figure 1:**
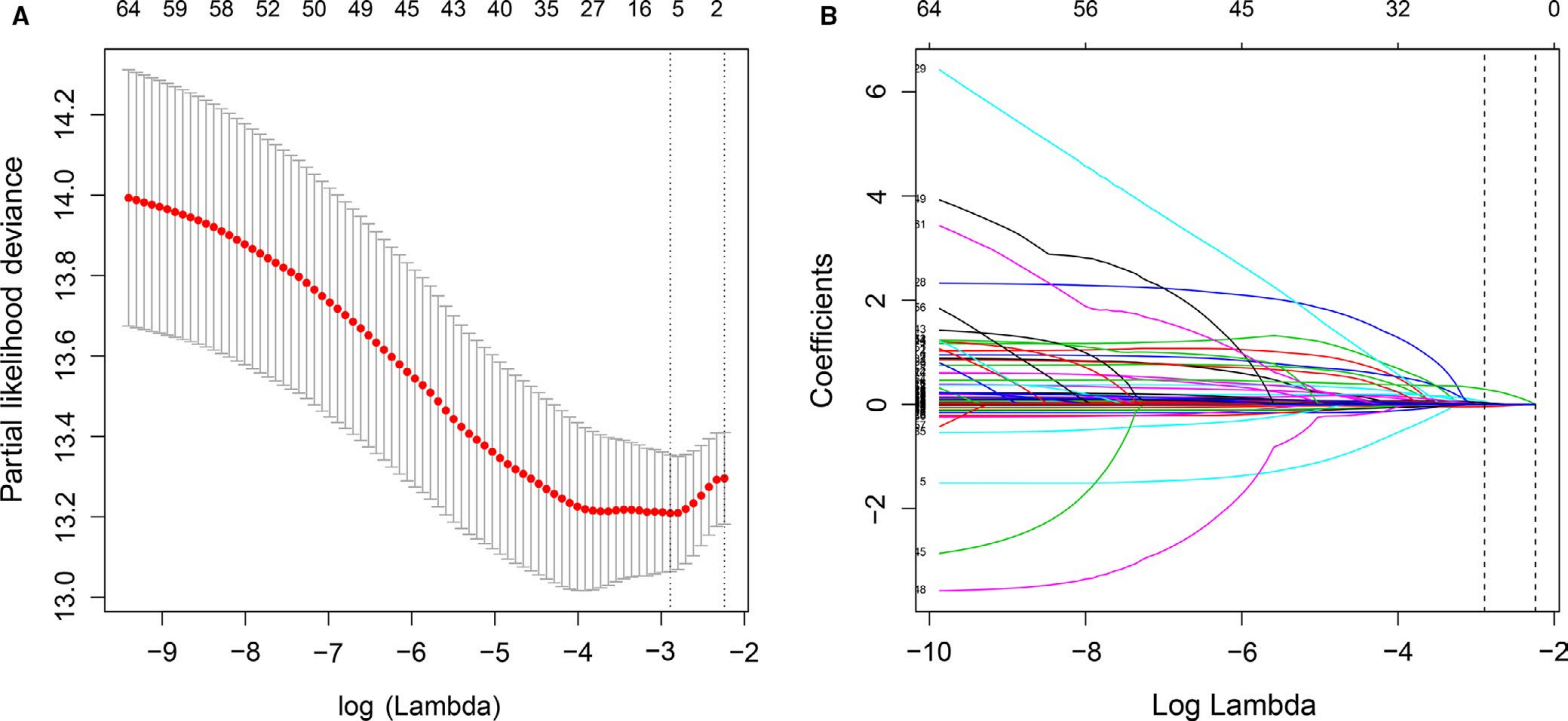
Feature selection using least absolute shrinkage and selection operator (LASSO) COX regression. A, Selection of tuning parameter (*λ*) in the LASSO regression using 10‐fold cross‐validation via minimum criteria. The partial likelihood binomial deviance is plotted vs log (*λ*). At the optimal values log (*λ*), where features are selected, dotted vertical lines are set using the minimum criteria and the one standard error of the minimum criteria. B, LASSO coefficient profiles for clinical features, each coefficient profile plot is produced vs log (λ) sequence. Dotted vertical line is set at the nonzero coefficients selected via 10‐fold cross‐validation, where six nonzero coefficients are included

In the univariate Cox regression analyses, 59 features were significantly related to OS. Among these, platelet distribution width was the feature most significantly associated with OS (*P* = 5.23 × 10^−11^). Additionally, sex, pathological T‐staging, N‐staging, M‐staging, and pathological stage, the *P*‐values of which were all > .05, were included. All 64 features and *P*‐values are listed in Table S2. These 64 features were considered as potential predictors. We applied a LASSO regression algorithm based on each feature for feature selection in the training cohort. The most appropriate tuning parameter *λ* for LASSO regression was 0.055 when the partial likelihood binomial deviance reached its minimum value (Figure 1A); six variables with nonzero coefficients were retained in the LASSO analysis (Figure 1B).

### 3.3 Construction of the nomogram and performance

**Figure 2:**
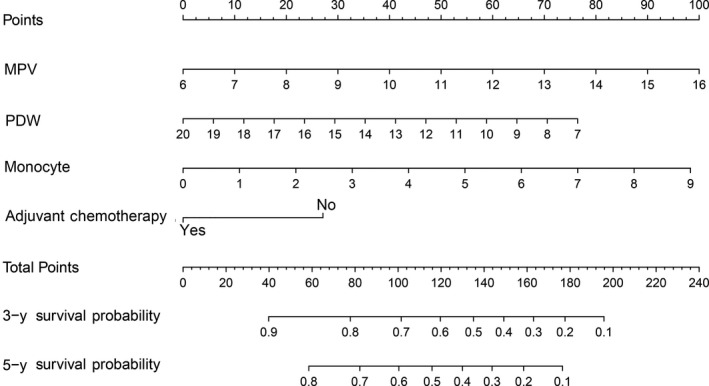
Four points are allocated for preoperative mean platelet volume, preoperative platelet distribution width, monocytes, and postoperative adjuvant chemotherapy. Nomogram for predicting 3‐ and 5‐year probabilities of colorectal cancer patients was established. Draw a vertical straight line from the variable value to the axis labeled “Points”. Then calculate all variables’ points. The total points on the bottom scales that correspond to the 3‐ and 5‐y survival were showed apparently

**Figure 3:**
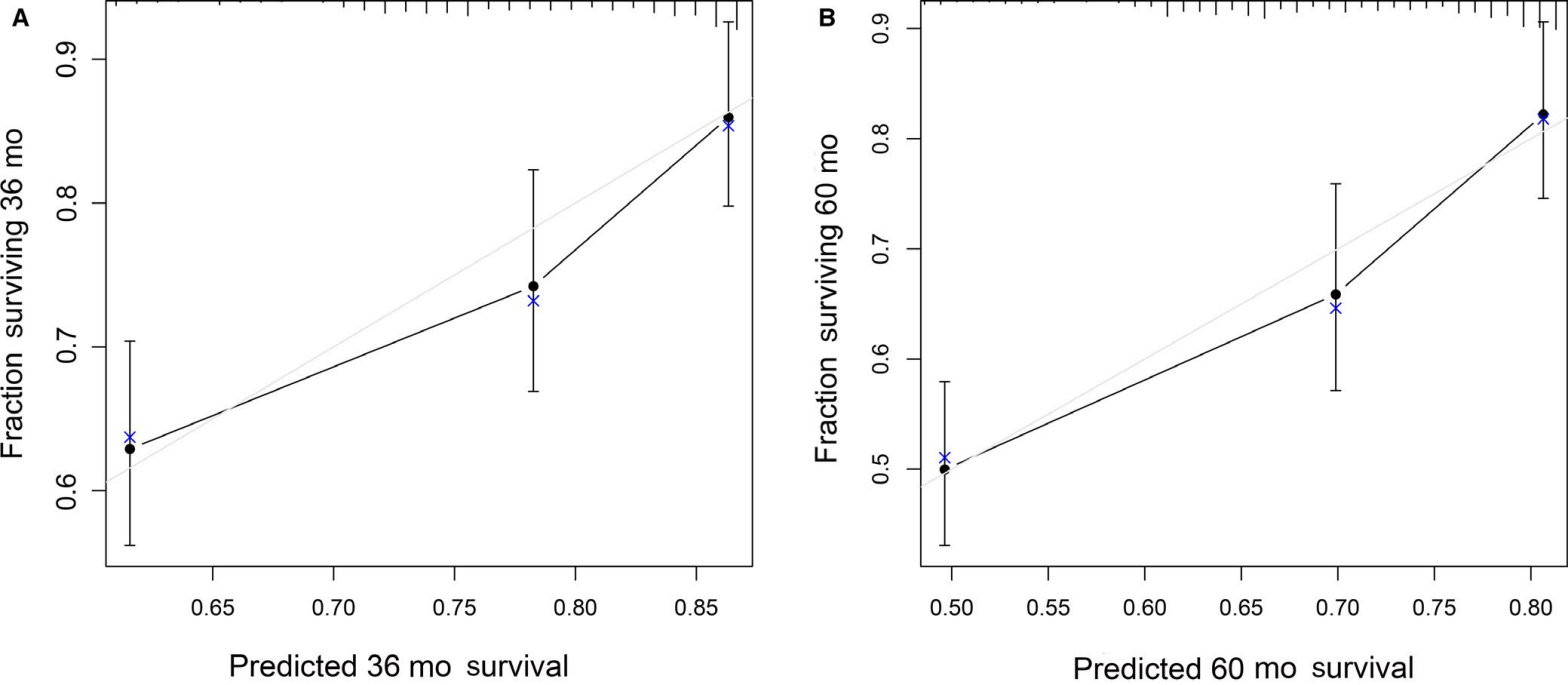
Calibration curves for predicting (A) 3‐y and (B) 5‐y OS in the training cohort. Predicted survival produced by nomogram is plotted on the x‐axis, and actual survival is plotted on the *y*‐axis. Dashed lines represent an identical calibration model in which predicted OS approximate to actual OS

The six retained variables were used for multivariate Cox proportional hazards analysis. Among these, preoperative mean platelet volume (MPV, *P* = .0047), preoperative platelet distribution width (PDW, *P* = .0006), monocytes (*P* = .0086), and postoperative adjuvant chemotherapy (*P* = .0011) were independent predictors for OS in patients with CRC. Thus, these four variables were selected for the construction of the nomogram. Multivariate Cox proportional hazards analysis was performed using the four selected variables. As shown in Table 2, these four variables were independent predictors of OS for patients with II‐III stage CRC. The nomogram was constructed using four respective variable point scales, with the sum of the four variable points defined as total points. By drawing a perpendicular line from the total point axis to the two outcome axis, estimated 3‐ and 5‐year survival probabilities could be obtained (Figure 2). The C‐index of the training set was 0.67, indicating good discrimination. To assess the calibration of the nomogram for the training set, we compared the predicted 3‐ and 5‐year survival probabilities to the actual 3‐ and 5‐year survival probabilities. As shown in Figure 3, the calibration curve revealed good concordance between the predicted and observed probabilities, and the modified Nam‐D'Agostino test yielded nonsignificant *P*‐values for both the 3‐ and 5‐year survival probabilities (3‐year, *P* = .55; 5‐year, *P* = .65). These results indicated that the nomogram had proper calibration for the training set.

**Table 2:** Multivariable cox regression analysis of the selected clinical features in the training set

Variable	Odds Ratio (95% CI)	*P*
Adjuvant Chemotherapy		.0011
Yes	1	
No	1.71 (1.24‐2.36)	
MPV	1.21 (1.06‐1.40)	.0047
PDW	0.89 (0.83‐0.95)	.0006
Monocytes	1.24 (1.06‐1.46)	.0086

### 3.4 Validation of the nomogram

**Figure 4:**
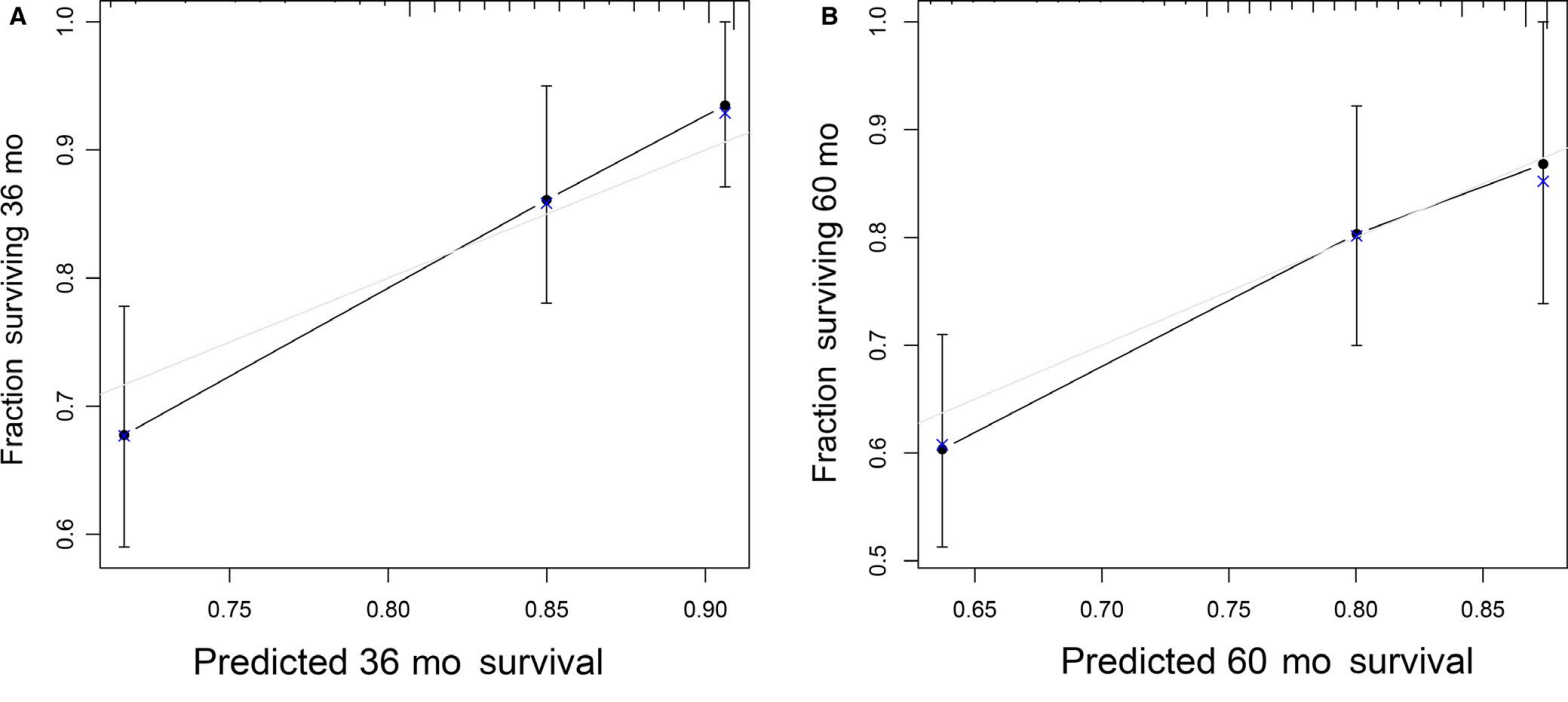
Calibration curves for predicting (A) 3‐y and (B) 5‐y OS in the validation cohort. Predicted survival produced by nomogram is plotted on the x‐axis, and actual survival is plotted on the *y*‐axis. Dashed lines represent an identical calibration model in which predicted OS approximate to actual OS

We next performed validation for the nomogram using the validation set. The C‐index of the validation cohort was 0.69, showing acceptable discrimination. The calibration curve revealed good concordance between the predicted and observed probabilities and modified Nam‐D'Agostino test yielded nonsignificant *P*‐values for both 3‐ and 5‐year survival probabilities (3‐year, *P* = .46; 5‐year, *P* = .80). These results indicated that the nomogram had proper calibration for the validation set (Figure 4).

### 3.5 Performance of the nomogram in risk stratification of patients

**Figure 5:**
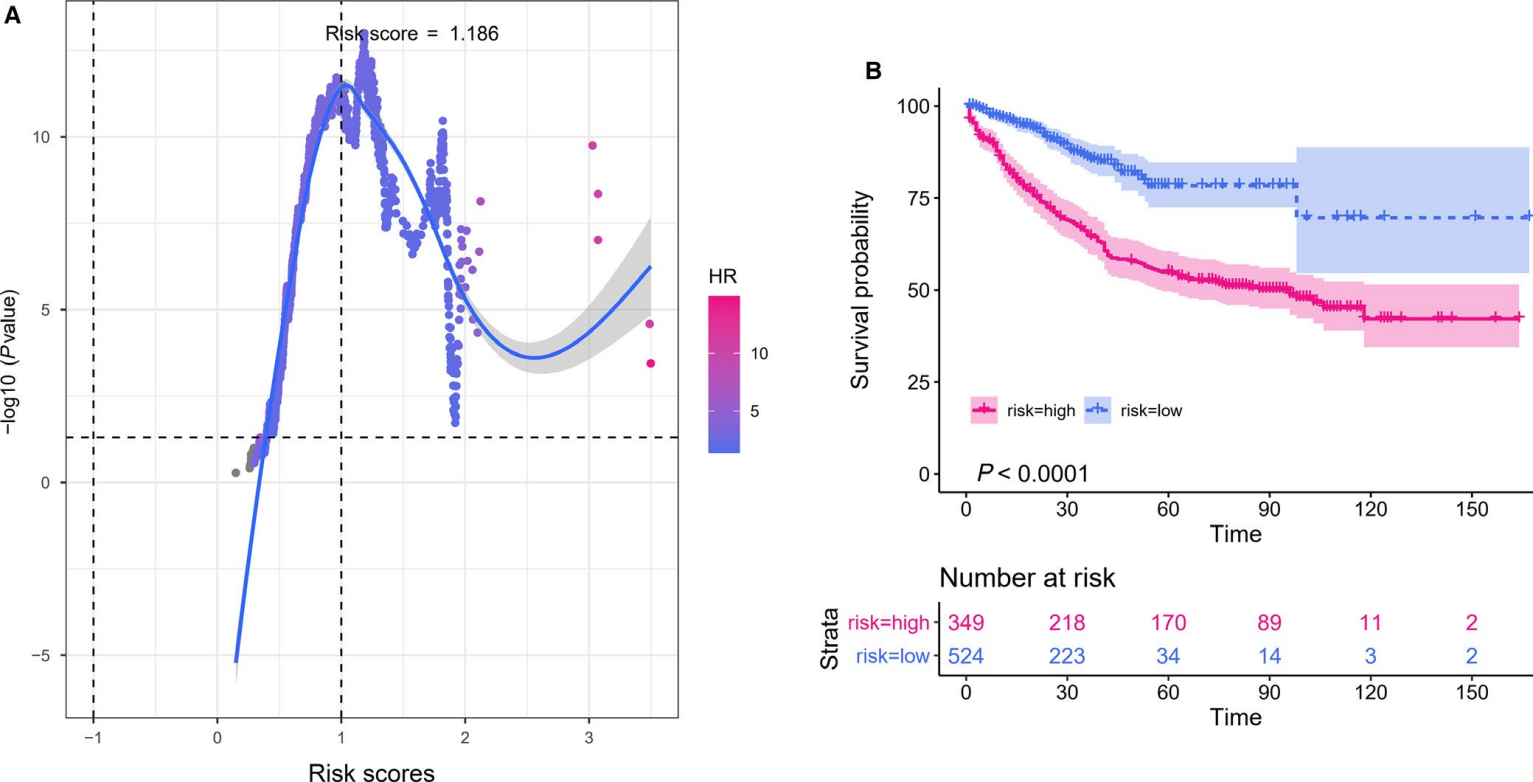
Kaplan‐Meier curves for overall survival of low‐risk group and high‐risk group based on the identified cutoff value. A, Determine the optimal segmentation threshold for dividing patients. Shown were the different risk scores and corresponding log‐rank *P*‐values. B, Kaplan‐Meier curves for overall survival of high‐risk patients and low‐risk patients based on the optimal segmentation threshold

We further calculated the risk scores of every patient based on the nomogram. To determine the optimal segmentation threshold for dividing patients into two subgroups (high‐risk and low‐risk groups), we repeatedly split the patients based on each risk score. As shown in Figure 5A, the minimum *P*‐value of the log‐rank test was obtained when the segmentation threshold was set at 1.186. Then, patients were divided into high‐risk and low‐risk groups based on the optimal segmentation threshold. Kaplan‐Meier curves were drawn for both groups; patients in the two different risk subgroups showed significant differences in OS (*P* < .001) (Figure 5B).

## 4 DISCUSSION

CRC is obviously diversified with regard to patient survival, even when all of the patients go through surgical resection and chemotherapy.^19^ Additional individualized treatment after surgical resection may offer improved OS in high‐risk patients with CRC. Therefore, the proper prognosis of patients with CRC deserves further research. At present, the individual survival of patients with CRC is usually predicted via the TNM staging system, which has limitations in its precision. In recent years, various prognostic models have been described,^20^, ^21^, ^22^, ^23^, ^24^, ^25^ but an ideal survival prediction model developed for stage II‐III CRC has not yet been created. Thus, intensive study regarding the prediction of individual survival for postoperative patients with CRC is urgently needed. Our research sought to construct and validate a model to calculate the long‐term survival of patients with operable CRC.

Our primary data were complete and well‐organized. Through the LASSO regression algorithm, which effectively processed the demographic and clinical feature selection as a statistical method for high‐dimensional data, we distinguished preoperative MPV, preoperative PDW, monocytes, and postoperative adjuvant chemotherapy as independent prognostic factors. Many scholars have reported that MPV levels might serve as potential biomarkers for the diagnosis and early recognition of different stages of CRC.^26^, ^27^ Furthermore, it was previously reported that patients with increased MPV had worse survival rates than those of patients with normal MPV levels.^28^ Additionally, one study found that the PDW levels of patients with CRC were higher than those in healthy participants but lower than those in adenomatous patients.^26^ That signified that PDW was an advantageous prognostic factor for the survival of patients with CRC. Furthermore, research has suggested that colorectal infection could induce an inflammatory reaction and promote CRC development.^29^ Therefore, we speculated that monocytes, which play an essential role in the inflammatory response, could be an independent prognostic factor. As independent prognostic factors, MPV, PDW, and monocytes are easily collected from routine blood tests. It has been proposed that these factors might help stratify patients with cancer into those who may or may not benefit from adjuvant chemotherapy.^28^ One study showed that systemic chemotherapy and regional chemotherapy reduced hematogenous metastasis in patients with CRC after resection, suggesting improved survival rates.^29^ Therefore, the inclusion of these four clinical features into our nomogram is consistent with the findings of these previous works.

We consolidated the four selected characteristics into our nomogram by multivariate analysis of the training set, then estimated its performance by calibration and discrimination. Both clinicians and patients alike could conveniently calculate the individual survival probability using this point‐based nomogram. On the basis of the total points and defined threshold values, we stratified patients with CRC into high‐risk and low‐risk groups. With regard to the high‐risk group, clinicians could provide rational suggestions for additional individualized therapy and intensive follow‐up. Validation is an essential procedure in nomogram studies to avoid poor goodness‐of‐fit and determine the generalizability of the model.^30^ In the present study, the calibration curve of the fraction surviving 36 months in the training set showed favorable agreement between the predicted and actual observed probabilities, and the fraction surviving 60 months showed more optimal agreement, which ensured the repeatability as well as reliability of the constructed model. Moreover, the nomogram indicated proper calibration in both the fraction surviving 36 months and the fraction surviving 60 months in the validation set as well. The C‐index of the training and validation sets were 0.67 and 0.69, respectively, revealing decent discriminatory capacity of the nomogram. Furthermore, the survival curves of these two risk groups had *P*‐values < .001, indicating satisfactory predictive performance.

Although some nomograms have been developed to predict individual survival probabilities for patients with CRC, there are some unique points in our nomogram. The tradeoff between comprehensiveness and comprehensibility can be challenging to balance and is unavoidable in nomogram studies. However, the applicable target of our nomogram is relatively comprehensive, involving stage II‐III CRC. In addition, improved nomogram accuracy often comes at the cost of increased complexity. Our nomogram is concise, with only four predictive factors, but remains accurate. All the clinical parameters needed for our nomogram are available after surgical resection and routine pathologic examination, without adding any burden to patients.

Though we successfully constructed and validated a nomogram to predict individual survival probability for patients with CRC after surgery resection, our study did have several limitations. First, our data were of limited size and from a single institution, which limit the generalizability and applicable scope of the nomogram. Secondly, there was no external validation from other institutions; single‐center data were used for external validation. Although the model still worked well in our internal cohort, which was intended for relatively strict validation, multi‐institutional external validation would provide more convincing evidence.

## 5 CONCLUSION

In summary, we constructed and validated an original predictive nomogram for the survival of patients with CRC after surgery, facilitating physicians to accurately evaluate the individual survival of postoperative patients and distinguish high‐risk patients who may need more aggressive treatment and follow‐up strategies.

## CONFLICT OF INTEREST

The author reports no conflict of interest in this work.

## AUTHOR CONTRIBUTIONS

Conceived and designed the experiments: WT, XH, JL, WY, XM; Performed the data collection: XH, JL, XM, GW, FJ, SC, CZ, WX, WT, WY, CL, ZL, LH, CT, JC, CW; Analyzed the data: XH, JL, XM, GW, WY, CL, ZL, LH, CT, JC, SC, CZ,CW; Contributed reagents/materials/analysis tools: XH, JL, XM, GW, FJ, SC, CZ, WT, WY, CL, ZL, LH, CT, JC, CW; Contributed to the writing of the manuscript: XH, JL, XM, WY, WT, CL, ZL, SC, CZ, LH, CT, JC, CW; All authors reviewed the manuscript.

## ETHICS APPROVAL AND CONSENT TO PARTICIPATE

This study was approved by the Ethics and Human Subject Committee of Guangxi Medical University Cancer Hospital.

## Data Availability

The dataset supporting the conclusions of this article is included within the article.
