# Supplementary material for: Nomogram for predicting overall survival in stage II‐III colorectal cancer
Source: Cancer Med. 2020 Feb 6;9(7):2363–71. doi: 10.1002/cam4.2896 (PMC7131840; doi:10.1002/cam4.2896)
Supplement: Supplementary file 1 [file CAM4-9-2363-s001.docx]

Supplementary table 1: Demographics and clinical features in the present study.

| Category | Features |
| --- | --- |
| General Information | Sex, age, weight, height |
| Diagnose | Clinical diagnosis: Rectal cancer  Clinical diagnosis: Rectosigmoid carcinoma  Clinical diagnosis: Sigmoid colon cancer  Clinical diagnosis: Descending colon cancer  Clinical diagnosis: Colonic splenic carcinoma  Clinical diagnosis: Transverse colon cancer  Clinical diagnosis: Colonic hepatic cancer  Clinical diagnosis: Ascending colon carcinoma  Clinical diagnosis: Ileocecal cancer  Clinical diagnosis: Multiple primary cancer |
| Symptom and sign | First symptoms: No symptom  First symptoms: Mucosanguineous feces  First symptoms: Stomachache  First symptoms: Change of stool habit  First symptoms: Thin stool  First symptoms: Anemia  First symptoms: Abdominal mass  First symptoms: Other  First symptoms: Change of stool character  First symptoms: Anal bulge  Onset time (month)  Loss of weight  Sore of ECOG  Tumor tenderness: Yes  Tumor tenderness: No  Tumor tenderness: Unknown |
| Previous history | Previous history: Colorectal cancer  Previous history: Nasopharyngeal carcinoma and head and neck tumors  Previous history: Breast cancer  Previous history: Esophagus cancer  Previous history: Gastric cancer  Previous history: Small intestinal carcinoma  Previous history: Hepatic carcinoma  Previous history: Pancreatic cancer  Previous history: Gynecological tumor  Previous history:Urinary system tumor  Previous history: Nervous system neoplasms  Previous history: Hemolymph system tumor  Previous history: Soft tissue sarcoma  Previous history: Other tumor  Previous history: None tumor  Basic disease: None  Basic disease: Diabetes  Basic disease: Hypertension  Basic disease: Coronary heart disease  Basic disease: Anemia  Basic disease: Hypoalbuminemia  Basic disease: Chronic obstructive pulmonary disease  Basic disease: Pneumonia  Basic disease: Asthma  Basic disease: Gout  Basic disease: Renal insufficiency  Basic disease: Tuberculosis  Basic disease: Hyperthyroidism  Basic disease: Arrhythmia  Basic disease: Other  Basic disease: Cerebral infarction  Basic disease: Gastritis  Basic disease: Gastric ulcer  Colorectal cancer related diseases: Ulcerative colitis  Colorectal cancer related diseases: Colorectal polyp  Colorectal cancer related diseases: Familial adenomatous polyposis  Colorectal cancer related diseases: Crohn's disease  Colorectal cancer related diseases: Schistosome  Colorectal cancer related diseases: Lynch syndrome  Colorectal cancer related diseases: Black spot polyposis  Colorectal cancer related diseases: None  Polyp location: Ileocecal region  Polyp location: Ascending colon  Polyp location: Transverse colon  Polyp location: Descending colon  Polyp location: Sigmoid colon  Polyp location: Rectum  Polyp location: None  Number of polyps |
| Family history | family history:Colorectal cancer  family history:Nasopharyngeal carcinoma and head and neck tumors  family history:Breast cancer  family history:Esophagus cancer  family history:Gastric cancer  family history:Small intestinal carcinoma  family history:Hepatic carcinoma  family history:Pancreatic cancer  family history:Gynecological tumor  family history:Urinary system tumor  family history:Nervous system neoplasms  family history:Hemolymph system tumor  family history:Soft tissue sarcoma  family history:Other tumor  family history:None tumor  family history:Lung cancer |
| Personal history | smoking history  alcohol drinking |
| Auxiliary examination | Blood type: A  Blood type: B  Blood type: AB  Blood type: O  Rh blood type: (+)  Rh blood type: (-)  Leukocyte  Hemoglobin  Blood platelet  Absolute number of monocytes  Absolute number of neutrophils  Absolute number of lymphocytes  Absolute number of eosinophils  Mean corpuscular volume  Red blood cell distribution width CV  Mean platelet volume (MPV)  Platelet distribution width (PDW)  Stool Routine  Urine protein  Urine occult blood  Total bilirubin  Direct bilirubin  Indirect bilirubin  Alanine aminotransferase (U/L)  Aspartate aminotransferase (U/L)  Preoperative alkaline phosphatase  Serum creatinine  Albumin  Globulin  Prealbumin  Total cholesterol  Triglyceride  Low-density lipoprotein cholesterol (mmol/l)  High-density lipoprotein cholesterol (mmol/l)  Apolipoprotein A  Apolipoprotein B  Fasting blood-glucose  Thymidine kinase 1 (TK1) cell cycle analysis  Heat shock protein 90  Preoperative CEA  Preoperative AFP  Preoperative ca-125  Preoperative ca-153  Preoperative ca-199  Total T lymphocytes  Helper lymphocytes  Inhibitory lymphocytes  TH/TS  Natural killer cell  B lymphocytes  lgG  lgM  lgA  C3  C4  CRP  HS-CRP  Neutrophil-lymphocyte ratio  Leukocyte on the first month postoperatively  Hemoglobin on the first month postoperatively  Blood platelet on the first month postoperatively  Blood glucose on the first month postoperatively  Alkaline phosphatase on the first month postoperatively  CEA on the first month postoperatively  AFP on the first month postoperatively  CA-125 on the first month postoperatively  CA-153 on the first month postoperatively  CA-199 on the first month postoperatively  NYHA:Ⅰ  NYHA:Ⅱ  NYHA:Ⅲ  NYHA:Ⅳ  ASA classification |
| Imaging and endoscopy examination | Distance from tumor to anus (cm)  Rectal tumor location  Rectal neoplasms occupy the lumen circumference  Whether the colonoscopy passed  Preoperative PET-CT Scanning  Preoperative MRI T staging  Preoperative MRI N staging  Preoperative MRI M staging  Preoperative CT T staging  Preoperative CT N staging  Preoperative CT M staging  Preoperative CT TNM staging |
| Pathological examination | Tumor differentiation  Gross type  Adenocarcinoma  Mucinous carcinoma  Other  Squamous carcinoma  Adenosquamous carcinoma  Signet-ring cell carcinoma  Total number of lymph nodes  Positive number of lymph nodes  Nerve invasion  Vascular invasion  Lymphatic invasion  CK7  Ck20  CDX-2  Villin  Ki67  Ki67（%）  S100  CD34  Microsatellite instability  MLH1  PMS2  MSH2  MSH6  Pathological: T-staging  Pathological: N-staging  M-staging  Pathological: stage  N-ras mutation: Exon 2(-)  N-ras mutation: Exon 3(-)  N-ras mutation: Exon 4(-)  N-ras mutation: Exon 2(+)  N-ras mutation: Exon 3(+)  N-ras mutation: Exon 4(+)  N-ras mutation: unknown  K-ras mutation: Exon 2(-)  K-ras mutation: Exon 3(-)  K-ras mutation: Exon 4(-)  K-ras mutation: Exon 2(+)  K-ras mutation: Exon 3(+)  K-ras mutation: Exon 4(+)  K-ras mutation: unkown  BRAF gene mutation  PI3K mutation: H1047R(-)  PI3K mutation: H1047L(-)  PI3K mutation: E542 K(-)  PI3K mutation: E545D(-)  PI3K mutation: E545K(-)  PI3K mutation: H1047R(+)  PI3K mutation: H1047L(+)  PI3K mutation: E542 K(+)  PI3K mutation: E545D(+)  PI3K mutation: E545K(+)  PI3K mutation: unknown |
| Postoperative situation | Postoperative exhaust time (h)  Postoperative feeding time (h)  Postoperative adjuvant chemoradiotherapy  Injury of anal function |
