# Supplementary material for: Nomogram for predicting overall survival in stage II‐III colorectal cancer
Source: Cancer Med. 2020 Feb 6;9(7):2363–71. doi: 10.1002/cam4.2896 (PMC7131840; doi:10.1002/cam4.2896)
Supplement: Supplementary file 2 [file CAM4-9-2363-s002.docx]

Supplementary table 2: Clinical features significantly associated with overall survival in univariate Cox regression analyses.

| ID | Features names | P-values |
| --- | --- | --- |
| 1 | Sex* | 0.924063411 |
| 2 | age | 0.045648272 |
| 3 | Previous history: Hemolymph system tumor | 0.007689012 |
| 4 | Clinical diagnosis: Rectosigmoid carcinoma | 0.008335929 |
| 5 | Clinical diagnosis: Colonic hepatic cancer | 0.005169676 |
| 6 | Polyp location: Ascending colon | 0.000441435 |
| 7 | Sore of ECOG | 0.00432915 |
| 8 | First symptoms: Anal bulge | 0.001825871 |
| 9 | Loss of weight | 0.002803119 |
| 10 | Tumor tenderness: Unknown | 0.00172632 |
| 11 | Preoperative PET-CT Scanning | 0.026481681 |
| 12 | Preoperative MRI T staging | 0.00476173 |
| 13 | Preoperative MRI N staging | 0.013189176 |
| 14 | Preoperative MRI M staging | 0.012651557 |
| 15 | Blood type: O | 0.014142261 |
| 16 | Absolute number of monocytes | 0.000830955 |
| 17 | Absolute number of neutrophils | 0.001245405 |
| 18 | Mean platelet volume (MPV) | 0.000189222 |
| 19 | Platelet distribution width (PDW) | 5.23E-11 |
| 20 | Urine occult blood | 0.047980973 |
| 21 | Preoperative ca-199 | 0.034459266 |
| 22 | C3 | 2.48E-05 |
| 23 | C4 | 0.013164974 |
| 24 | Neutrophil-lymphocyte ratio | 0.009185601 |
| 25 | NYHA:Ⅰ | 0.005674452 |
| 26 | Tumor differentiation | 0.000380901 |
| 27 | Gross type | 0.007579703 |
| 28 | Other | 0.015550875 |
| 29 | Total number of lymph nodes | 0.003939212 |
| 30 | Positive number of lymph nodes | 0.02363674 |
| 31 | Nerve invasion | 0.013260208 |
| 32 | CK7 | 9.70E-07 |
| 33 | Ck20 | 4.62E-07 |
| 34 | Villin | 3.14E-07 |
| 35 | Ki67 | 0.026454089 |
| 36 | S100 | 1.10E-06 |
| 37 | CD34 | 0.048435675 |
| 38 | Microsatellite instability | 3.82E-08 |
| 39 | MLH1 | 9.05E-08 |
| 40 | PMS2 | 1.12E-07 |
| 41 | MSH2 | 1.19E-07 |
| 42 | MSH6 | 1.31E-07 |
| 43 | Pathological:T-staging* | 0.761937285 |
| 44 | Pathological:N-staging* | 0.424409019 |
| 45 | M-staging* | - |
| 46 | Pathological: stage* | 0.424409019 |
| 47 | N-ras mutation: Exon 2(-) | 0.004566437 |
| 48 | N-ras mutation: Exon 3(-) | 0.014869184 |
| 49 | N-ras mutation: Exon 4(-) | 0.014291786 |
| 50 | N-ras mutation: unkown | 0.014498809 |
| 51 | K-ras mutation: Exon 3(-) | 0.028226519 |
| 52 | K-ras mutation: Exon 4(-) | 0.022021222 |
| 53 | K-ras mutation: unkown | 0.014664314 |
| 54 | BRAF gene mutation | 6.43E-08 |
| 55 | PI3K mutation: H1047R(-) | 0.015071858 |
| 56 | PI3K mutation: H1047L(-) | 0.01137026 |
| 57 | PI3K mutation: E542 K(-) | 0.012645493 |
| 58 | PI3K mutation: E545D(-) | 0.012645493 |
| 59 | PI3K mutation: E545K(-) | 0.0078657 |
| 60 | PI3K mutation: unkown | 0.02072562 |
| 61 | Postoperative exhaust time (h) | 0.03515285 |
| 62 | Postoperative feeding time (h) | 0.018860244 |
| 63 | Postoperative adjuvant chemoradiotherapy | 1.86E-06 |
| 64 | Injury of anal function | 0.037232844 |

*: P-value >0.05
